# Regulations of Gene Expression in Medullary Thymic Epithelial Cells Required for Preventing the Onset of Autoimmune Diseases

**DOI:** 10.3389/fimmu.2013.00249

**Published:** 2013-08-26

**Authors:** Taishin Akiyama, Miho Shinzawa, Junwen Qin, Nobuko Akiyama

**Affiliations:** ^1^Division of Cellular and Molecular Biology, Institute of Medical Science, University of Tokyo, Tokyo, Japan; ^2^Key Laboratory for Regenerative Medicine, Department of Developmental and Regenerative Biology, Ministry of Education and International Base of Collaboration for Science and Technology, the Ministry of Science and Technology and Guangdong Province, Jinan University, Guangzhou, China

**Keywords:** medullary thymic epithelial cells, autoimmune disease, NF-κB, TNF receptor family, gene expression

## Abstract

Elimination of potential self-reactive T cells in the thymus is crucial for preventing the onset of autoimmune diseases. Epithelial cell subsets localized in thymic medulla [medullary thymic epithelial cells (mTECs)] contribute to this process by supplying a wide range of self-antigens that are otherwise expressed in a tissue-specific manner (TSAs). Expression of some TSAs in mTECs is controlled by the autoimmune regulator (AIRE) protein, of which dysfunctional mutations are the causative factor of autoimmune polyendocrinopathy-candidiasis-ectodermal dystrophy (APECED). In addition to the elimination of self-reactive T cells, recent studies indicated roles of mTECs in the development of Foxp3-positive regulatory T cells, which suppress autoimmunity and excess immune reactions in peripheral tissues. The TNF family cytokines, RANK ligand, CD40 ligand, and lymphotoxin were found to promote the differentiation of AIRE- and TSA-expressing mTECs. Furthermore, activation of NF-κB is essential for mTEC differentiation. In this mini-review, we focus on molecular mechanisms that regulate induction of AIRE and TSA expression and discuss possible contributions of these mechanisms to prevent the onset of autoimmune diseases.

## Introduction

The thymus contributes to self-tolerance of T cells by eliminating potentially self-reactive T cells and generating immunosuppressive T cells, which are essential for preventing the onset of autoimmune disease. Epithelial cells localized in the thymic medulla [medullary thymic epithelial cells (mTECs)] are non-hematopoietic in origin and play non-redundant roles in the elimination of self-reactive T cells (1–4). Recent studies have revealed that mTECs also contribute to the selection and survival of immunosuppressive Foxp3-positive regulatory T cells (Tregs) (5–8).

Medullary thymic epithelial cells express several functional molecules required for the selection of self-tolerant T cells and Tregs (3). Mature types of mTECs express MHC molecules and co-stimulatory molecules essential for antigen presentation to developing T cells. In addition, mTECs secrete several types of chemokines (e.g., CCL19, CCL21, and CCL22) that attract T cells or dendritic cells in the medulla (2, 9). Moreover, a recent study has shown that the expression of CD70 in mTECs enhances the development and survival of Tregs via an interaction with its receptor, CD27, which is expressed on thymic T cells (5). A key feature of mTECs is their ability to express hundreds of self-antigens that are normally expressed in a tissue-specific manner (TSAs) (4, 10). TSAs are processed and directly presented by mTECs or indirectly presented by thymic DCs receiving TSAs from mTECs (4, 7, 11–13). T cells that recognize TSAs with high avidity undergo apoptosis (so-called negative selection) or survive as regulatory T cells (4, 14). Many studies have suggested significant roles of mTEC-dependent self-tolerance in preventing the onset of some autoimmune diseases in humans. Expression of some TSAs requires a nuclear protein autoimmune regulator (AIRE), the dysfunctional mutations of which are responsible for an inherited human autoimmune disease, autoimmune polyendocrinopathy-candidiasis-ectodermal dystrophy (APECED) (15, 16). Whereas the expression of AIRE mRNA is detected in different cell types, AIRE expression at the protein level is remarkably high in mTECs (17). A previous study using AIRE-deficient mice provided evidence that autoimmunity, provoked by dysfunction of AIRE, is thymic stroma-dependent (18). In addition to APECED, recent studies have demonstrated that single-nucleotide polymorphisms (SNPs) in the *AIRE* gene are associated with rheumatoid arthritis (19, 20). In addition to mutations in the *AIRE* gene, reduced expression of the muscle acetyl choline receptor (*CHRNA1*) in mTECs was shown to be associated with the onset of myasthenia gravis (21). Moreover, impairment of the mTEC-dependent tolerance might explain the relationship between myocarditis and autoimmunity (22). These findings also imply that the onsets of various human autoimmune diseases could be related to dysregulation of mTEC-dependent tolerance. Interestingly, in addition to relationships with autoimmune diseases, recent studies have uncovered roles for mTEC-dependent T-cell tolerance in tumor tolerance (8, 23, 24).

Because expression of AIRE and TSAs is characteristic of mTEC, mTECs should harbor specific mechanisms to direct AIRE and TSA expression. Expression of TSAs appears to be correlated with the differentiation of mTECs. In this mini-review, we specially focus on molecular mechanisms regulating the expression of AIRE and TSAs and the process of mTEC differentiation.

## Development of mTECs

Thymic epithelial cells are classified into mTECs and cortical thymic epithelial cells (cTECs) (2). Several lines of evidence indicate the existence of a bi-potent TEC progenitor capable of differentiating into mTECs and cTECs in the fetal and adult thymus (25–29). The bi-potent TEC progenitor seems to give rise to each progenitor of mTECs and cTECs in the next stage (30, 31). Recent studies revealed that mTECs differentiate from progenitors expressing cTEC-markers (32, 33). These data imply that mechanisms determining the mTEC commitment suppress the cTEC-driving program. However, master molecules that decide the fate of the bi-potent TEC progenitor expressing cTEC-markers to the mTEC lineage have not been determined yet.

**Figure 1:**
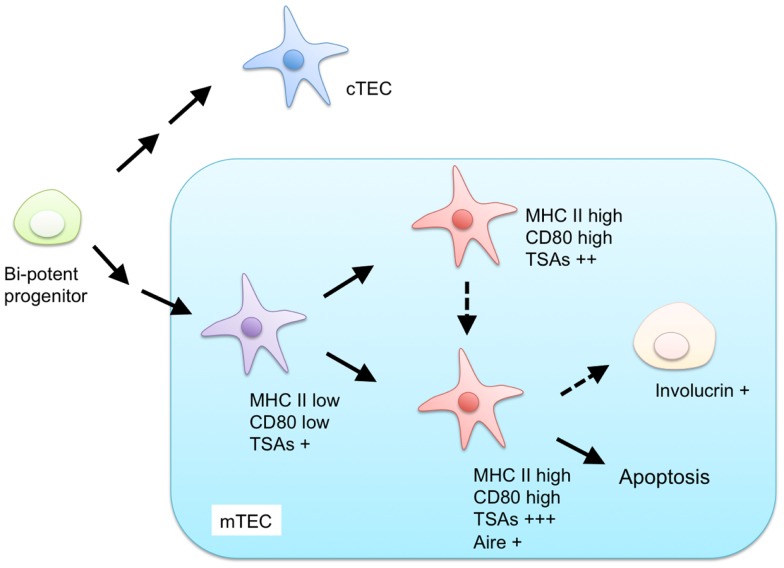
**Proposed model for differentiation of mTECs**. Both mTECs and cTECs are generated from a bi-potent progenitor in the fetal and adult thymus. mTECs are classified by expression of MHC class II (MHC II), CD80, AIRE, and involucrin. mTECs expressing low levels of MHC II and CD80 are considered immature and give rise to mature mTECs, expressing high levels of MHC II and CD80, and a more diverse set of tissue-specific antigens (TSAs). MHC II-high and CD80-high mature mTECs are further separated into AIRE-positive and AIRE-negative subpopulations. AIRE-positive mature mTECs are postmitotic and undergo apoptosis or otherwise differentiate into involucrin-positive mTECs.

Currently, mTECs are classified based on the expression of MHC II, CD80, AIRE, and involucrin (Figure 1). mTECs (typically defined as CD45^−^ EpCAM^+^ Ly51^−^ and UEA-1^+^ by flow cytometric analysis) in adult mice are divided into two subpopulations, according to the expression levels of MHC II and CD80 (34). mTECs expressing high levels of MHC II and CD80 (mTEC^hi^) express a more diverse set of TSAs than mTECs expressing lower levels of MHC II and CD80 (mTEC^lo^) do (35). Moreover, precursor-product relationship analysis has suggested that the mTEC^lo^ fraction can differentiate into mTEC^hi^ (36, 37). Therefore, the mTEC^hi^ fraction would be the more mature type of mTEC than mTEC^lo^.

The mTEC^hi^ fraction is further separated on the basis of AIRE expression (36, 38). Because previous studies have indicated that the AIRE-expressing mTECs^hi^ (AIRE^+^ mTEC^hi^) are postmitotic and susceptible to apoptosis (36), AIRE^+^ mTECs^hi^ are postulated to be the more differentiated cell types than AIRE-negative mTECs^hi^. mTECs expressing involucrin, a marker of terminally differentiated keratinocytes, are considered to be terminally differentiated mTECs that may be derived from AIRE^+^mTEC^hi^ (39, 40).

## Regulation of AIRE mRNA Expression

Molecular mechanisms regulating the expression of AIRE, which are likely critical for preventing autoimmunity, remain unclear. In the fetal thymus, expression of AIRE starts at embryonic day 14.5 (41). Consistently, mature mTECs emerge around this embryonic day (42). Thus, AIRE expression seems to be closely linked to mTEC differentiation. However, because mTEC^hi^ is separated into AIRE^+^ and AIRE^-^fractions, the mTEC differentiation mechanism might be necessary but is not entirely sufficient for AIRE expression.

A study using a luciferase reporter assay identified a plausible minimal promoter region of the *AIRE* gene (43). This region contains binding sequences for Sp1, AP-1, NF-Y, and ETS family of transcription factors. Indeed, luciferase reporter analysis suggested regulation of the *AIRE* gene promoter by ETS family proteins (44). However, *in vivo* genetic studies are necessary to prove that these sequence-specific transcription factors are critical for the regulation of AIRE expression.

The promoter region of AIRE contains a high ratio of CpG sites (43). These CpG sites are hypermethylated in established cell lines defective in the AIRE expression. A subsequent study showed that these CpG sites are hypomethylated in isolated mTECs compared to thymocytes (45). These findings suggest that DNA demethylation might be prerequisite for AIRE expression. However, interestingly, hypomethylation was also observed in cTECs and thymoma with defective AIRE expression (45). Hence, DNA hypomethylation appears to be required but not sufficient for inducing AIRE expression.

Overall, AIRE expression seems to be regulated by combinations of chromatin modification and sequence-specific transcription factors. However, precise mechanisms and regulatory molecules remain to be determined.

## Regulation of TSA mRNA Expression

TSA expression appears to be regulated by complicated mechanisms. Single-cell PCR analyses revealed a stochastic nature of TSA expression in mTECs (38, 46). Each TSA is expressed in a subset of mTECs (38, 46). The frequency of mTECs expressing a particular TSA was different, depending on the TSA (38, 46). Interestingly, various combinations of TSAs are expressed in individual mTECs (38, 46). These studies suggest that regulatory mechanisms of TSA expression in mTECs are different from those used in inherent tissues.

Several studies suggest that TSA expressions are epigenetically controlled. A comprehensive mRNA expression study revealed that TSA gene loci tend to co-localize in chromosomal clusters (35, 47). Moreover, genomic imprinting of the *Igf2* gene, a TSA, was lost in mTECs (35), implicating the involvement of a DNA demethylation mechanism in TSA expression. Interestingly, another imprinted gene, *Cdkn1c*, was not affected. These data imply the existence of mTEC-specific mechanisms for demethylation of DNA.

Control of TSA gene expression by AIRE has been intensively studied (48–50). Several studies have revealed a function of AIRE as a transcription factor that directly promotes TSA expression (51, 52). Furthermore, AIRE binds to hypomethylated Histone 3 Lys 4 (H3K4) through its plant homology domain (53, 54). This finding suggests that AIRE modifies the chromatin structure in the TSA genes. AIRE also binds to DNA-PK (55–57), which functions in the repair of DNA-double strand breakage. A study using an mTEC cell line suggested that interactions of AIRE with H3K4 and DNA-PK are critical in recruiting AIRE to TSA gene loci and promoting TSA expression (57). Additionally, it was reported that AIRE interacts with P-TEFb, a component of the super elongation complex (58). It is generally accepted that transcription elongation, via the release of “paused” RNA polymerase II, is critical for the regulation of many genes (58, 59). AIRE may recruit P-TEFb to the TSA gene locus and promote elongation of the arrested TSA transcripts by releasing RNA polymerase II from the proximal promoter (60). Recent comprehensive analysis of mRNA transcripts in mTECs supports this mechanism (61). In addition to the TSA expression, the AIRE-dependent expression of some microRNAs (miRNAs) was recently revealed (62, 63). Consistently, genetic studies revealed important roles played by miRNA expressions in functions and maintenance of mTECs (63–65).

Compared to the mechanisms underlying Aire-dependent TSA expression, molecular mechanisms underlying Aire-independent TSA expression are less understood. As described above, whereas epigenetic regulations of TSA genes would be critical, mechanisms underlying epigenetic changes specific for mature mTECs remain unclear. Moreover, unidentified transcription factors may be involved in the promotion of Aire-independent TSA expressions.

## Extracellular Signaling to Promote Differentiation of mTECs Expressing AIRE and TSAs

Differentiation of TECs is well known to be correlated to differentiation of T cells in the thymus (so-called thymic cross-talk) (3). mTEC maturation was reported to be abolished in severe combined immunodeficiency (SCID) patients (66). This finding supports the idea that failure of the thymic cross-talk results in the onset of autoimmune manifestation through inhibition of mTEC function. Interestingly, a recent study showed that administration of anti-CD3ε antibody ameliorated autoimmunity in leaky SCID model mice possibly through improvement of the thymic cross-talk (67).

Molecular basis of the thymic cross-talk in mTEC development has been reported. Several lines of evidence revealed that TNF family cytokines expressed in thymocytes and other cells of hematopoietic origin (2) and their receptors expressed in mTEC are critical for the thymic cross-talk. Briefly, signaling of TNF receptor family members, RANK, CD40, and lymphotoxin-β receptor (LtβR), play essential roles in the development of mTECs expressing Aire and TSAs. This topic has been summarized in a recent review (1).

## Downstream of TNF Receptor Family Signaling

TNF receptor family signaling induces the activation of NF-κB and MAPK pathways (68). To date, the involvement of the MAPK pathway in the development of mTEC remains to be addressed. However, several lines of evidence have indicated that the NF-κB family plays a critical role in the development of mTECs expressing AIRE and TSAs.

**Figure 2:**
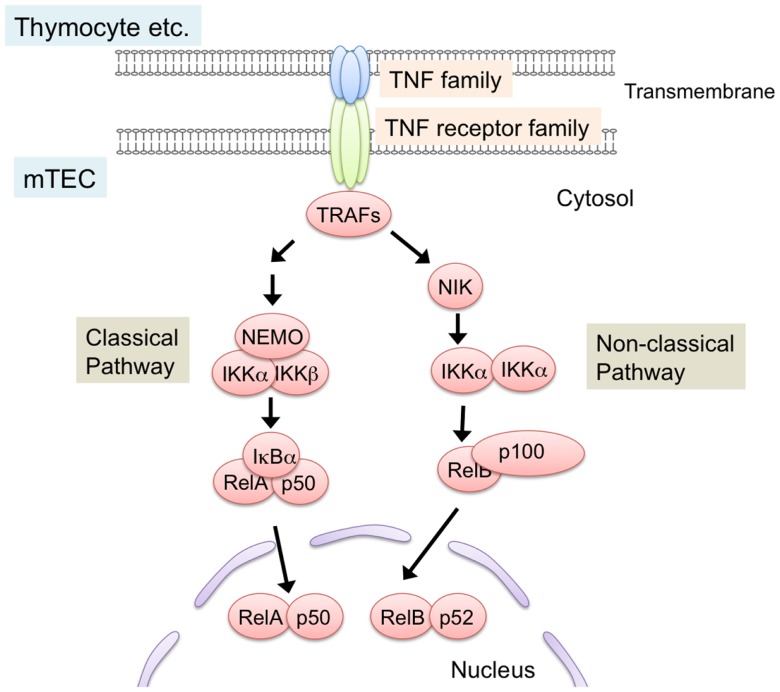
**NF-κB activation pathways triggered by TNF family signaling**. Interaction of TNF family ligand (RANK ligand, CD40 ligand, and lymphotoxin α and β complex) with their respective receptors (RANK, CD40, and LtβR) induces activation of NF-κB pathways. Interaction between the ligand and its receptor induce the binding of TRAF-family proteins to the cytoplasmic domains of TNF receptors. TRAF-family proteins in turn activate downstream serine/threonine kinase cascade. These kinases trigger the degradation of inhibitory proteins that sequester NF-κB in cytosol, thereby leading to the translocation and transcriptional activation of NF-κB members. NF-κB pathways are classified into classical and non-classical pathway. In the non-classical pathway, NF-κB complex consisting of RelB and p52 is activated. NIK is critical for the non-classical NF-κB pathway. TRAF6, a member of the TRAF protein family, was reported to regulate only the classical NF-κB pathway, which causes nuclear translocation of mainly the RelA complex. On the other hand, other TRAF members function in the non-classical NF-κB pathway by binding to the TNF family receptors.

NF-κB members are sequestered in the cytoplasm in an inactive state by the binding of the inhibitory protein IκB in resting cells (69–71). Ligations of receptors induce phosphorylation and subsequent degradation of IκB proteins, thereby leading to nuclear localization of NF-κB to activate transcription. Two distinct NF-κB activation pathways, the classical pathway and the non-classical pathway, are currently known (70–72) (Figure 2). The classical pathway is required in inflammatory responses and lymphocyte activation (71). On the other hand, the non-classical pathway mainly promotes development and architecture formation of lymphoid organs, including the thymus. In the non-classical pathway, receptor ligation induces accumulation of the NF-κB-inducing kinase (NIK), which is normally degraded by the ubiquitin-dependent proteasome in resting cells. Subsequently, accumulated NIK phosphorylates and activates IKKα, which induces partial degradation of p100 to p52. p100 preferentially binds to and sequesters RelB in the cytoplasm, and the partial degradation of p100 to p52 induces translocation of RelB and p52 as a heterodimer into the nucleus.

The requirement for NF-κB activation in the development of mTEC was initially identified by the analysis of RelB-deficient mice (73, 74). RelB-deficient mice showed severe reduction in medulla size, accompanied by a lack of UEA-1-positive mTECs. Consistently, the expression of AIRE was abolished in the RelB-deficient thymus (6, 41, 75). As expected, RelB-deficient mice showed severe autoimmune diseases. A recent study demonstrated that autoimmunity of RelB mice was due to the defect in thymic stroma function (6). Mice carrying a dysfunctional mutation, NIK (*aly/aly*), also showed a similar defect in mTEC development and autoimmune phenotypes (76–78). Whereas IKKα-deficient mice die shortly after birth, neonatal IKKα-deficient mice and transplantation of IKKα-deficient thymic stroma indicates a requirement of IKKα in the development of mTECs (79, 80). mTEC development in p100-deficient mice is partially defective (81, 82), but this appears to be due to a partial rescue of p100 function by p105 (or its processed product, p50) because the double deficiencies of p100 and p105 resulted in severe defects in mTEC development, similar to the RelB- and NIK-mutant mice (83). Overall, these results support the idea that activation of the non-classical NF-κB pathway is essential for the development of mTECs.

TRAF6 is a signal transducer that mediates signaling from TNF receptor family members (84, 85). TRAF6-deficient mice exhibit severe autoimmune disease (86, 87). Additionally, recent studies suggest possible associations between SNPs of the *TRAF6* gene with rheumatoid arthritis and systemic lupus erythematosus in humans (88, 89). Previous studies showed that TRAF6 promotes the development of mTECs expressing AIRE and TSAs, thereby suppressing autoimmunity (86). Moreover, RANK-mediated differentiation of mTECs requires TRAF6 in *in vitro* organ culture of fetal thymic stroma (90). Notably, TRAF6 is a signal transducer that mediates the activation of the classical NF-κB pathway but not the non-classical NF-κB pathway (84, 85). Thus, these data imply a role for TRAF6-mediated activation of the classical NF-κB pathway in mTEC differentiation.

In addition to the above findings, a scaffold protein, Sin (also called Efs), was proposed to be expressed downstream of TNF receptor family signaling. Sin-deficient mice showed reduced numbers of mTECs and thymic stroma-dependent autoimmunity (91). In addition to the role of Sin in FGF-mediated proliferation signaling (91), a recent study suggested that Sin might regulate the non-classical NF-κB pathway activated by RANKL signaling (92). Because the SH3 domain and phosphorylation of tyrosine residues of Sin might be critical for its function (93, 94), these studies also imply unrecognized roles of Src-type tyrosine kinases in mTEC development.

## Concluding Remarks

Whereas significant roles for NF-κB in signal activation of mTEC differentiation and subsequent expression of AIRE and TSAs are indisputable, molecular events connecting these signaling pathways to induction of AIRE and TSA remain unclear. It was reported that LtβR signaling induces the expression of AIRE in an mTEC line in the presence of a DNA methylation inhibitor (95). However, it is still unclear whether NF-κB binds to the promoter of the *AIRE* gene. Moreover, a wide variety of TSA expression would not be explained only by NF-κB-dependent transcriptional activation because NF-κB family members are generally known to be sequence-specific transcription factors. Thus, the link between NF-κB activation and expression of AIRE and TSAs remains largely enigmatic.

In addition, differentiation stages regulated by these signaling molecules and their mechanisms need to be clarified. mTECs have different properties in each developmental stage, with regard to TSA expression, AIRE expression, and DNA methylation status. Therefore, it is important to clarify types of mTECs in which each TNF receptor family signal functions. Overall, more studies are needed to understand the molecular and cellular mechanisms regulating the development of mTECs with the final aim to develop novel therapeutic strategies preventing autoimmune diseases caused by defective thymic functions.

## Conflict of Interest Statement

The authors declare that the research was conducted in the absence of any commercial or financial relationships that could be construed as a potential conflict of interest.
